# *Salmonella enterica* serovar Infantis from Food and Human Infections, Switzerland, 2010–2015: Poultry-Related Multidrug Resistant Clones and an Emerging ESBL Producing Clonal Lineage

**DOI:** 10.3389/fmicb.2017.01322

**Published:** 2017-07-13

**Authors:** Denise Hindermann, Gopal Gopinath, Hannah Chase, Flavia Negrete, Denise Althaus, Katrin Zurfluh, Ben D. Tall, Roger Stephan, Magdalena Nüesch-Inderbinen

**Affiliations:** ^1^Institute for Food Safety and Hygiene, University of Zurich Zürich, Switzerland; ^2^Center for Food Safety and Applied Nutrition, U.S. Food and Drug Administration, Laurel MD, United States

**Keywords:** *Salmonella* Infantis, Hungarian clone B, *bla*_CTX-M-65_, food, humans

## Abstract

**Objectives:** The aim of this study was to characterize a collection of 520 *Salmonella enterica* serovar Infantis strains isolated from food (poultry meat), human infections and environmental sources from the years 2010, 2013 and 2015 in Switzerland.

**Methods:** We performed antimicrobial susceptibility testing and pulsed-field gel electrophoresis (PFGE) analysis on all 520 *S*. Infantis isolates, and whole genome sequencing (WGS) on 32 selected isolates.

**Results:** The majority (74.8%) of the isolates was multidrug resistant (MDR). PFGE analysis revealed that 270 (51.9%) isolates shared an identity of 90%. All isolates subjected to WGS belonged to sequence type (ST) 32 or a double-locus variant thereof (one isolate). Seven (21.9%) of the sequenced isolates were phylogenetically related to the broiler-associated clone B that emerged in Hungary and subsequently spread within and outside of Europe. In addition, three isolates harboring *bla*_CTX-M-65_ on a predicted large (∼320 kb) plasmid grouped in a distinct cluster.

**Conclusion:** This study documents the presence of the Hungarian clone B and related clones in food and human isolates between 2010 and 2015, and the emergence of a *bla*_CTX-M-65_ harboring MDR *S*. serovar Infantis lineage.

## Introduction

Non-typhoidal *Salmonella enterica* (NTS) are one of the most important etiological agents of foodborne diarrheal diseases in humans worldwide and cause an estimated 80.3 million foodborne illnesses a year (Majowicz et al., 2010). Although most cases of salmonellosis are self-limiting episodes of gastro-enteritis, severe cases of infection, including bacteremia and meningitis require antimicrobial treatment. Ciprofloxacin is a common first-line antimicrobial for treating salmonellosis but because fluoroquinolones are not used for treating children, β-lactams (ampicillin or third-generation cephalosporins) are of equal importance (Medalla et al., 2016). Multidrug-resistant (MDR) NTS is associated with higher morbidity and mortality outcomes compared to drug-susceptible strains and is a major public health concern (Mølbak, 2005). *Salmonella enterica* subsp. *enterica* serovar Infantis (*S*. serovar Infantis) has emerged as the fourth most common serovar causing human salmonellosis in Europe, with 1,846 cases reported by the EU/EEA countries in 2014 (European Food Safety Authority [EFSA] and European Centre for Disease Prevention [ECDC], 2016). Poultry, especially from layer and broiler farms, as well as pigs are the main animal reservoirs for *S.* serovar Infantis (Nógrády et al., 2012). This serovar is also dominant in broiler meat, accounting for 35.9% of all *Salmonella* isolates reported from EU countries in 2014 (European Food Safety Authority [EFSA] and European Centre for Disease Prevention [ECDC], 2016). Over the last few years, antimicrobial resistance has emerged in *S*. serovar Infantis isolates from human and animal sources in various European countries and consequently, this serovar, together with *S*. Kentucky, contributes significantly to the numbers of MDR *Salmonella* in Europe (Nógrády et al., 2008; Dionisi et al., 2011). Closely related MDR clones of *S*. serovar Infantis have disseminated among broiler populations and associated animal growing environments, ultimately being disseminated into the food chain and then into humans in European countries such as Hungary, Poland and Austria (Nógrády et al., 2012). Isolates belonging to these clones are characterized by their resistance to nalidixic acid, sulfamethoxazole, streptomycin and tetracycline (NaSSuT). Recently, resistance to third generation cephalosporins has emerged in *S*. serovar Infantis isolates in Italy, due to the circulation of an extended-spectrum β-lactamase (ESBL) producing, MDR clone with additional reduced susceptibility to ciprofloxacin (Franco et al., 2015). The spread of MDR *S*. serovar Infantis clones throughout the food production system (mainly poultry and poultry meat) and in humans is highly worrisome and warrants improved understanding of its epidemiology. In Switzerland, *S*. serovar Infantis ranks among the top five of *Salmonella* serovars registered by the National Centre for Enteropathogenic Bacteria and Listeria (NENT). However, currently no data on antimicrobial resistance patterns or clonal relationships of the isolates exist, despite its clinical importance.

The aim of this study was to characterize a collection of 520 *S*. serovar Infantis strains isolated from food (poultry meat), human infections and environmental sources from the years 2010, 2013 and 2015 in Switzerland (i) by determining their phenotypic antibiotic resistance profiles using the disk diffusion method and (ii) by assessing genotypic characteristics and clonal relatedness using molecular methods including pulsed-field gel electrophoresis (PFGE), PCR, and whole genome sequencing (WGS).

## Materials and Methods

### Bacterial Strains

A total of 520 non-duplicate *S*. serovar Infantis isolates from human infections (*n* = 84), poultry meat (*n* = 418) and other sources (*n* = 18) were collected during 2010, 2013 and 2015 at the National Centre for Enteropathogenic Bacteria and Listeria (NENT), Switzerland. The isolates had been forwarded by hospitals, diagnostic laboratories or surveillance programs of retail markets and food or feed producing facilities for final species-level identification according to the White-Kauffmann-Le Minor scheme (Grimont and Weill, 2008).

### Antimicrobial Susceptibility Testing

Antimicrobial susceptibility testing was performed using the disk-diffusion method and the antibiotics ampicillin (AM), amoxicillin-clavulanic acid (AMC), cefotaxime (CTX), nalidixic acid (Na), ciprofloxacin (CIP), gentamicin (GM), kanamycin (K), streptomycin (S), sulfamethoxazole (Su), trimethoprim (TMP), tetracycline (T), and chloramphenicol (C) (Becton Dickinson, Heidelberg, Germany). Results were interpreted according to Clinical and Laboratory Standards Institute (CLSI) performance standards (CLSI, 2016). For sulfamethoxazole, for which breakpoints are not listed separately from trimethoprim, an inhibition zone of ≤10 mm was interpreted as resistant. Isolates displaying resistance to three or more classes of antimicrobials (counting β-lactams as one class) were defined as multidrug-resistant (MDR). Synergistic effects between AMC and CTX were regarded as an indication of the presence of an ESBL producer (Kaur et al., 2013).

### Detection and Characterization of Extended-Spectrum β-Lactamase (*bla*) Genes

Putative ESBL producers were grown on Brilliance^TM^ESBL agar (Oxoid, Hampshire, United Kingdom). The presence of *bla*_ESBLs_ was confirmed by PCR by screening for *bla*_TEM_, *bla*_SHV_, and *bla*_CTX-M_ alleles belonging to CTX-M groups 1, 2, 8, 9, and 25 as described previously (Woodford et al., 2006; Geser et al., 2012; Zurfluh et al., 2015). Synthesis of primers and DNA custom sequencing was carried out by Microsynth (Balgach, Switzerland) and nucleotide sequences were analyzed with CLC Main Workbench 6.6.1. For database searches the BLASTN program of NCBI^1^ was used.

### Pulsed-Field Gel Electrophoresis

Pulsed-field gel electrophoresis was performed according to the PulseNet protocol of the Centers for Disease Control and Prevention (CDC)^2^, using the restriction enzyme *XbaI* (Roche, Mannheim, Germany) and *Salmonella* serovar Braenderup strain H9812 (ATCC BAA 664) as the reference strain. Restricted DNA was separated in a 1% agarose gel (BioRad, Cressier, Switzerland) at 12°C for 20 h at 6 V/cm under linear ramp with switch times from 2 to 64 s and 120° included angle using a CHEF-DR III system (BIO-RAD, Munich, Germany). Gels were stained with ethidium bromide and visualized under UV light with Gel Doc (BIO-RAD, Munich, Germany). GelCompar II software (Applied Maths NV, Sint-Martens-Latem, Belgium) was used for analysis. Pairwise similarities between the *XbaI* PFGE patterns were calculated by the DICE similarity coefficient. Clustering was based on the unweighted pair-group method with averages, setting tolerance at 1% and optimization at 0.5%.

### Whole Genome Sequencing

Whole genome sequencing was performed with a representative subset of 32 isolates selected with regard to their PFGE pattern, source, year of isolation or presence of *bla*_CTX-M-65_. DNA extraction was performed with the Wizard^®^ Genomic DNA Purification Kit according to the manufacturers protocol (Promega AG, Dübendorf, Switzerland). Sequencing was performed on a MiSeq sequencer (Illumina, San Diego, CA, United States), utilizing a 600 cycle Nextera XT library kit. Trimmed Fastq data sets were *de novo* assembled using the recommended workflow on CLC Genomics Work bench version 8.0 (CLC bio, Aarhus, Denmark). Genomic contigs were annotated using the RAST annotation server (Aziz et al., 2008). The assembled sequences were uploaded to the http://www.genomicepidemiology.org/ server. Sequences of the seven housekeeping genes (*aroC, dnaN, hemD, hisD, purE, sucA* and *thrA*) were analyzed to identify multilocus sequence types (MLST)^3^, antibiotic resistance genes^4^, and plasmid replicon types^5^, using each website’s algorithms and databases. Routine processing of genome datasets was carried out by in-house perl scripts (available upon request). A local customized database of *Salmonella* chromosomal and plasmid genomes from NCBI was created and used for annotation, plasmid analysis, and homology detection with BLAST suite (Altschul et al., 1990). Phylogenetic analysis was conducted using a multiloci analysis based on a published core gene dataset (Leekitcharoenphon et al., 2012). Alleles in 2780 core gene loci across all the genomes were identified with *Salmonella enterica* subsp. *enterica* serovar Typhimurium LT2 genome as the reference. Of these loci, 1,500 core gene loci were randomly chosen and alleles were concatenated. The data matrix of alleles was subject to multiple alignment and phylogenetic analysis using tools available in MEGA7 suite (Kumar et al., 2016). The phylogenetic tree was built using the Neighbor-Joining algorithm in the MEGA 7 suite with 9,562 positions across 50 genomes for the final analysis.

For genome comparison, whole genome draft sequences of *Salmonella enterica* serovar Infantis strains from public databases were retrieved, including four recent strains of the prevalent Hungarian clone B of *S*. serovar Infantis from Hungary (Wilk et al., 2016, 2017), five *bla*_ESBL_ harboring, and eight non-*bla*_ESBL_ harboring strains from Italy, Israel and the United States. (Aviv et al., 2014; Franco et al., 2015), and one fully susceptible strain from the United Kingdom (Olasz et al., 2015). An overview of the strains and their GenBank accession numbers is given in **Table 1**. For plasmid comparison, annotations were obtained from RAST (Aziz et al., 2008), and compared with pCSAM042077 (Tate et al., 2017) on the SEED server for additional verification (Overbeek et al., 2013).

**Table 1:** *S*. Infantis strains used for genome comparison in this study.

Strain	Alias	Year of isolation	Region of isolation	Sample type	Source	GenBank accession no.	Reference
SI3337/12	3337 12 H	2012	Hungary	Animal	Broiler	MIJS00000000	Wilk et al., 2016
SI757/13	757 13 H	2013	Hungary	Animal	Broiler	MIJT00000000	Wilk et al., 2016
SI786/13	786 13H	2013	Hungary	Animal	Broiler	MIJR00000000	Wilk et al., 2016
SI1070/16	1070 16	2016	Hungary	Animal	Broiler	MRUX00000000	Wilk et al., 2017
13017779/5	brochM113	2013	Italy	Animal	Broiler	ERR1014114	Franco et al., 2015
12037823/11	brchM112	2012	Italy	Animal	Broiler	ERR1014117	Franco et al., 2015
13002124/1	huM12013	2013	Italy	Clinical	Human	ERR1014112	Franco et al., 2015
14026835	italy 2014	2014	Italy	Clinical	Human	ERR1014119	Franco et al., 2015
CFSAN042077	CFSAN042077	2015	United States	Food	Broiler	FSIS1502916	Tate et al., 2017
120100	120100	2008	Israel	Food	Unknown	SAMN04217889	Aviv et al., 2016
119944	119944	2008	Israel	Clinical	Human	ASRF01000000	Aviv et al., 2014
07041415	broMESC07	2007	Italy	Food	Broiler	ERR1014109	Franco et al., 2015
09051564/79	broMESC09	2009	Italy	Animal	Broiler	ERR1014118	Franco et al., 2015
FDA00001200	CFSAN014765	2008	Israel	Food	Basil	SRR3453168	CFSAN^a^
06029746	bromIEs6	2006	Italy	Food	Broiler	ERR1014111	Franco et al., 2015
335 3	335 3 israel	1970	Israel	Clinical	Human	SAMN02470973	Aviv et al., 2016
09051564/33	guifoIEc9	2009	Italy	Animal	Guinea fowl	ERR1014110	Franco et al., 2015
1326/28	infan649235	1973	United Kingdom	Animal	Broiler	SAMEA3106395	Olasz et al., 2015

^a^*U.S. Food and Drug Administration’s Center for Food Safety and Applied Nutrition*.

## Results

### Antimicrobial Susceptibility Testing

The distribution of resistance phenotypes among the *S*. serovar Infantis isolates is summarized in the **Table 2** and shown in detail in technical Supplementary Tables S1–S3.

**Table 2:** Origin and antimicrobial resistance characteristics among *S*. Infantis in Switzerland.

		No. isolates with			
	No. isolates analyzed	resistance pattern		resistance to	
Year/Source		NaSSuT^a^	NaSuT^b^	AMP^c^	CIP^d^
2010	191				
Food	167	64 (38.3%)	72 (43.1%)	4 (2.4%)	3 (1.8%)
Human	19	2 (10.5%)	5 (26.3%)	0 (0%)	0 (0%)
Other	5	2 (40%)	0 (0%)	0 (0%)	0 (0%)
2013	161				
Food	131	64 (48.9%)	32 (24.4%)	3 (2.3%)	7 (5.3%)
Human	27	8 (29.6%)	8 (29.6%)	3 (11.1%)	1 (3.7%)
Other	3	0 (0%)	0 (0%)	0 (0%)	0 (0%)
2015	168				
Food	120	31 (25.8%)	57 (47.5%)	6 (5%)	13 (10.8%)
Human	38	14 (36.8%)	12 (31.6%)	4 (10.5%)	0 (0%)
Other	10	4 (10%)	4 (10%)	0 (0%)	0 (0%)

^a^*NaSSuT, nalidixic acid-streptomycin-sulfamethoxazole-tetracycline resistance pattern. ^b^NaSuT, nalidixic acid-sulfamethoxazole-tetracycline resistance pattern. ^c^AMP, ampicillin. ^d^CIP, ciprofloxacin*.

**FIGURE 1:**
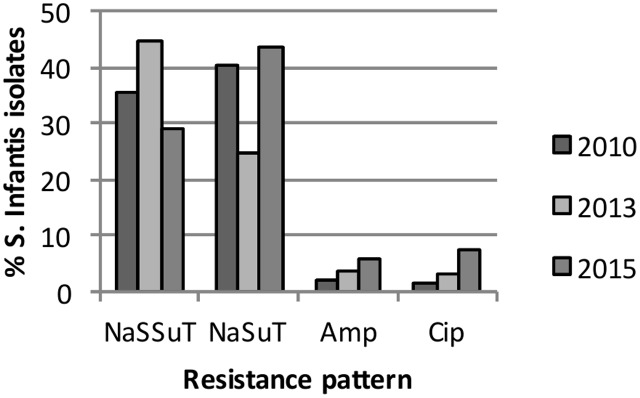
Percentage of *S*. serovar Infantis isolates with the nalidixic acid-sulfamethoxazole-tetracycline (NaSuT), or the nalidixic acid-streptomycin-sulfamethoxazole-tetracycline resistance pattern (NaSSuT) resistance pattern and resistance to ampicillin and ciprofloxacin in 2010, 2013 and 2015.

Multidrug resistant was detected in 389 (74.8%) of the isolates. Thereof, the majority (379/520, 72.9% of all isolates) showed a combined resistance pattern to nalidixic acid, sulfamethoxazole and tetracycline, with (NaSSuT) or without streptomycin (NaSuT). The NaSSut pattern was detected in 189 (36.3% of all isolates) and the NaSuT pattern in 190 (36.5% of all isolates). Resistance to ampicillin and ciprofloxacin was verified in 20 (3.8%) and 24 (4.6%) of the isolates, respectively, and showed a rising prevalence between 2010 and 2015 for both antimicrobials, as illustrated in **Figure 1**. A total of 496 (95.4% of all isolates) tested within the intermediate range for ciprofloxacin, according to CLSI breakpoints. An ESBL phenotype and growth on Brilliance^TM^ESBL agar was recorded for three strains (0.6% of all isolates), whereof two originated from humans (isolates 21-13 and 125-15) and one (isolate 144-13) from food (**Table 3** and Supplementary Tables S2, S3).

**Table 3:** Characteristics of 32 sequenced *S*. Infantis from food, diseased humans and other sources from 2010, 2013 and 2015 from Switzerland.

Strain ID	Year of isolation	Source	Phenotypic resistance pattern^a^	Resistance genes detected by WGS	ST	PFGE cluster	Accession no.
17-10	2010	Food	NaSSuT	*aadA1*, *sul1*, *tetA*	32	–	NAPL00000000
78-10	2010	Human	NaSSuT	*aadA1*, *sul1*, *tetA*	32	C	NKQL00000000
79-10	2010	Human	NaSSuT	*aadA1*, *sul1*, *tetA*	32	A	NAPI00000000
111-10	2010	Other	Su	none	32	–	NAPF00000000
115-10	2010	Food	NaSuT	*aadA1*, *sul1*, *tetA*, *bla*_TEM-116_	32	–	NAPD00000000
169-10	2010	Food	NaSuT	*aadA1*, *sul1*, *tetA*	32	B	NAOU00000000
186-10	2010	Human	NaSuT	*aadA1*, *sul1*, *tetA*	32	D	NAOS00000000
193-10	2010	Food	NaSSuT	*aadA1*, *sul1*, *tetA*	32	–	NAOR00000000
21-13	2013	Human	NaSSuT, AMP, CTX	*aph4-la*, *aadA1*, *aac3-IVa*, *aph3′-lc*, *bla*_CTX-M-65_, *fosA*, *floR*, *sul1*, *tetA*, *dfrA14*	32 variant^b^	–	NAPP00000000
25-13	2013	Food	NaSSuT	*aadA1*, *sul1*, *tetA*	32	D	NAPO00000000
31-13	2013	Food	NaSSuT	*aadA1*, *sul1*, *tetA*	32	–	NAPN00000000
53-13	2013	Human	NaSuT	*aadA1*, *sul1*, *tetA*	32	E	NAPV00000000
61-13	2013	Food	Su	none	32	–	NAPJ00000000
100-13	2013	Food	none	none	32	–	NAPU00000000
112-13	2013	Food	NaSuT	*aadA1*, *sul1*, *tetA*	32	–	NAPE00000000
123-13	2013	Food	NaSuT	*aadA1*, *sul1*, *tetA*	32	C	NAPB00000000
133-13	2013	Food	NaSuT	*aadA1*, *sul1*, *tetA*	32	–	NAOY00000000
144-13	2013	Food	NaSSuT, AMP, CTX	*aph4-la*, *aadA1*, *aac3-IVa*, *aph3′-lc*, *bla*_CTX-M-65_, *fosA*, *floR*, *sul1*, *tetA*, *dfrA14*	32	–	NAOX00000000
							
153-13	2013	Human	Na, Su	*aadA1*, *sul1*	32	–	NAOV00000000
173-13	2013	Human	NaSSuT	*aadA1*, *sul1*, *tetA*	32	C	NBAS00000000
3-15	2015	Human	NaSSuT	*aadA1*, *sul1*, *tetA*	32	B	NAPW00000000
15-15	2015	Human	NaSSuT	*aadA1*, *sul1*, *tetA*	32	A	NAPM00000000
20-15	2015	Human	NaSuT	*aadA1*, *sul1*, *tetA*	32	–	NAPK00000000
69-15	2015	Human	NaSuT	*aadA1*, *sul1*, *tetA*	32	–	NAPH00000000
70-15	2015	Other	NaSuT	*aadA1*, *sul1*, *tetA*, *floR*, *dfrA14*	32	–	NKQM00000000
97-15	2015	Human	NaSSuT	*aadA1*, *sul1*, *tetA*	32	–	NAPG00000000
99-15	2015	Human	None	None	32	–	NJAM00000000
119-15	2015	Other	NaSSuT	*aadA1*, *aph3’-lc*, *sul1*, *tetA*, *dfrA14*	32	–	NAPC00000000
125-15	2015	Human	NaSuT, AMP, CTX	*aph4-la*, *aadA1*, *aac3-IVa*, *bla*_CTX-M-65_, *fosA*, *floR*, *sul1*, *tetA*, *dfrA14*	32	–	NAPA00000000
126-15	2015	Human	None	None	32	–	NAOZ00000000
149-15	2015	Food	NaSuT	*aadA1*, *sul1*, *tetA*	32	–	NAOW00000000
169-15	2015	Food	NaSSuT	*aadA1*, *sul1*, *tetA*	32	C	NAOT00000000

^a^*NaSSuT, nalidixic acid-streptomycin-sulfamethoxazole- tetracycline resistance pattern; NaSuT, nalidixic acid-sulfamethoxazole-tetracycline resistance pattern; ^b^double-locus variant (*purE* 288 and *sucA* 565) of ST32. AMP, ampicillin; CTX, cefotaxime; Na, nalidixic acid; PFGE, pulsed-field gel electrophoresis; Su, sulfamethoxazole; ST, sequence type; WGS, whole genome sequencing; –, isolate not belonging to any specific PFGE cluster. Resistance genes: *aadA1*, *aph3′-lc*, *aac3-IVa*, *aph4-la*, aminoglycoside; *bla*, β-lactam; *fosA*, fosfomycin; *floR*, phenicol; *sul1*, sulphonamide; *tetA*, tetracycline; *dfrA14*, trimethoprim*.

### WGS

The genomic characteristics of the 32 genomes are listed in Supplementary Tables S1–S3. The average genome size, %G+C, and number of coding DNA sequences (CDS) of the isolates was 4.88 mbp (range:4.56–4.98 mbp), 52.2% (range: 52.1–52.3%), and 4812 CDS (range: 4443–4912 CDS), respectively.

### Detection of Resistance Genes by PCR and by WGS

All three isolates displaying an ESBL phenotype showed the presence of the *bla*_CTX-M-65_ gene by PCR. WGS analysis using ResFinder confirmed the presence of the *bla*_CTX-M-65_ genes. All *bla*_CTX-M-65_-positive isolates carried other resistance genes *aph4-la*, *aadA1* and *aac3-IVa* (aminoglycoside resistance), *fosA* (fosfomycin resistance), *floR* (phenicol resistance), *sul1* (sulphonamide resistance), *tetA* (tetracycline resistance), and *dfrA14* (trimethoprim resistance) (**Table 3** and Supplementary Tables S2, S3), while strains 21-13 and 144-13 also carried the *aph3′-lc* gene (aminoglycoside resistance). WGS of 23 MDR, non-ESBL producing isolates revealed the presence of *aadA1*, *sul1*, and *tetA* resistance genes throughout. Food isolate 115-10 additionally carried the non-ESBL *bla*_TEM-116_ gene. The environmental isolate 70-15 from Israel additionally harbored *floR* and *dfrA14* and MDR strain 119-15 isolated from animal feed also carried *aph3′-lc* and *dfrA14* (**Table 3** and Supplementary Table S3).

Whole genome sequencing did not detect any resistance genes in six non-MDR isolates (**Table 3** and Supplementary Tables S1–S3), except for human isolate 153-13 which showed intermediate resistance to streptomycin and harbored *aadA1* and *sul1* (**Table 3** and Supplementary Table S2).

### Detection of Plasmids and Replicon Types among the Isolates

**FIGURE 2:**
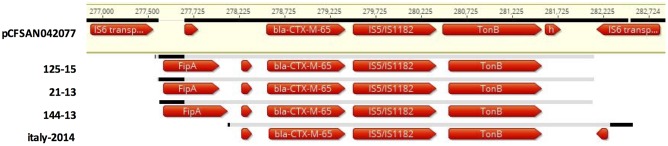
Multiple sequence alignment with pCSAM042077 from *S*. Infantis from food from the United States (FSIS150291), of a *bla*_CTX-M-65_ cassette on putative ∼320 kb plasmids from three *S*. Infantis strains from food (144-13) and diseased humans (21-13 and 125-15) from Switzerland and one *S*. Infantis strain from Italy (ERR1014119). The ∼5 kb cassette consists of *bla*_CTX-M-65_ followed by an IS5/IS1182 family transposase and a gene encoding a TonB-dependent siderophore receptor, and one or two hypothetical genes. The cassette is flanked on either side by the IS6 transposase. The *fipA* gene is of varied lengths, or absent.

By mapping WGS assemblies to the 320 kb plasmid pCFSAN42077 from *bla*_CTX-M-65_ carrying food isolate CFSAN42077 (Tate et al., 2017), contigs or regions similar to this plasmid in isolates 21-13, 144-13 and 125-15 were detected (data not shown). Specific contigs bearing the *bla*_CTX-M-65_ gene and surrounding loci were identified (**Figure 2**). The ∼5 kb cassette consisted of *bla*_CTX-M-65_ followed by an IS5/IS1182 family transposase and a gene encoding a TonB-dependent siderophore receptor. The 5′-end is flanked by an IS6 family transposase upstream of the *fipA* gene, which is truncated in pCFSAN42077. By comparison, *fipA* is absent in p14026835, a *bla*_CTX-M-65_ harboring plasmid from human *S*. Infantis isolate 14026835 from Italy (ERR1014119), as shown in **Figure 2**. As predicted, no replicons were detected in the genomes of these three *bla*_CTX-M-65_ harboring isolates (Franco et al., 2015).

For other isolates in this study, WGS analysis showed the presence of incompatibility group IncFII (p96A) plasmid (accession #JQ418521), in the fully susceptible human isolate 99-15 (N15-1280), Incl1 (accession #AP005147) in fully susceptible food isolate 100-13 (N13-1368), and pESA2 and IncFII (pCTU2) (accession nos. #CP000784 and #FN543095, respectively) in fully susceptible human isolate 126-15 (N15-1729) (data not shown).

### PFGE Cluster Analysis, MLST and Phylogenetic Analysis

#### PFGE

Pulsed-field gel electrophoresis analysis revealed 190 distinct *XbaI* restriction patterns (all patterns are available upon request). Of the 520 isolates, 270 (51.9%) shared a similarity of 90%. Five clusters A–E showing a similarity of >93% and consisting of 121 (23.3% of total) isolates were detected. Isolates within a cluster had indistinguishable profiles. The clusters A–E contained 21 (4% of the total), 18 (3.5%), 27 (5.2%), 43 (8.3%) and 12 (2.3%) isolates, respectively. Isolates from sources other than human infections or food (e.g., environmental samples) were not detected within these clusters.

#### MLST and Phylogenetic Analysis

All 32 isolates subjected to WGS belonged to sequence type (ST) ST32, except isolate 21-13, which was a double-locus variant (*purE* and *sucA*) of ST32 (**Table 3**).

**FIGURE 3:**
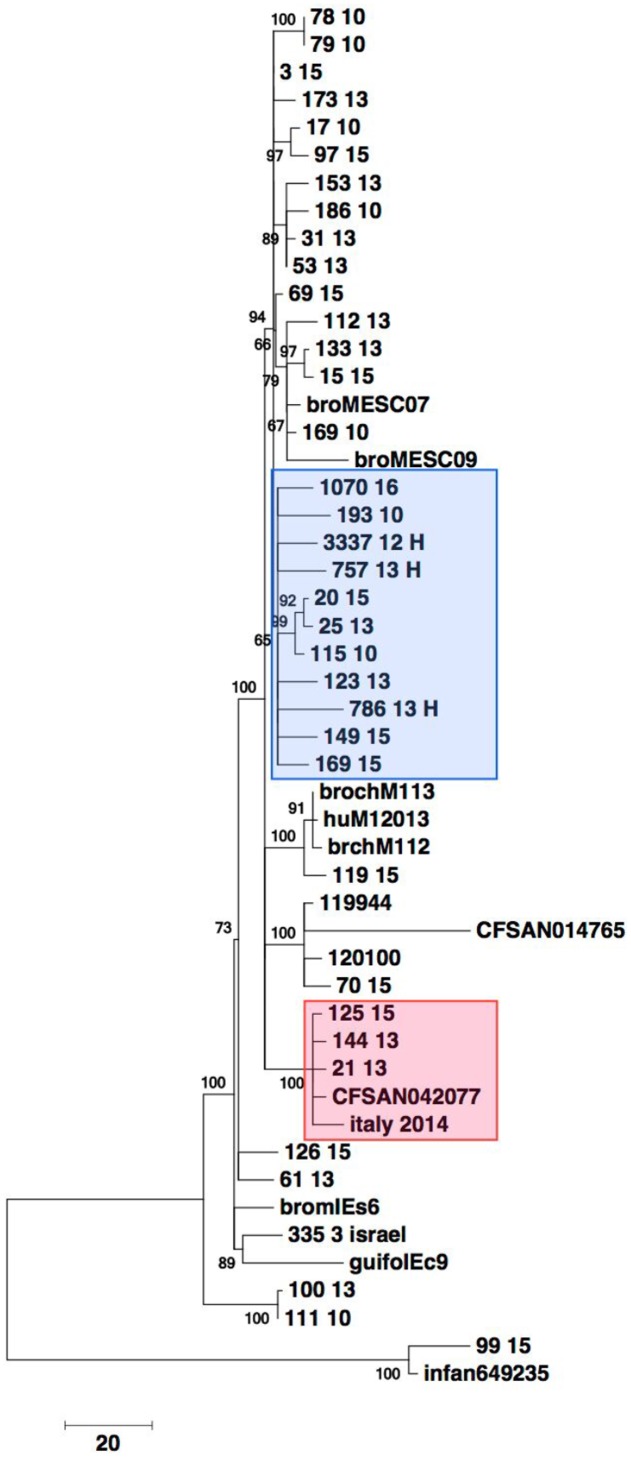
Core gene analysis based phylogeny of 32 selected *S*. serovar Infantis from food, diseased humans and environmental sources from 2010, 2013 and 2015 from Switzerland and of *S*. serovar Infantis from Hungary, Israel, Italy and United States. Alleles from 1500 randomly chosen core genes from more than 2770 conserved loci were used to build a phylogenetic tree. The data matrix of alleles was subject to multiple alignments. The phylogenetic tree was built using the Neighbor-Joining algorithm in the MEGA 7 suite with 9,562 positions across 50 genomes for the final analysis. Bar indicates 20 single nucleotide polymorphisms (SNP). Blue box: Isolates belonging to the Hungarian clone. Red box: Isolates harboring *bla*_CTX-M-65_.

Genetic relatedness was investigated by mapping the 32 genomes to the genome sequences of isolates belonging to the Hungarian clone B from broilers, the *bla*_ESBL_, and non-*bla*_ESBL_ harboring strains from Italy, Israel and United States. The resulting unrooted phylogenetic tree is shown in **Figure 3**. The isolates segregated into eight major clusters. Clusters determined by PFGE and by WGS did not correlate.

Close phylogenetic relatedness with strains 3337 12 H, 757 13 H, 786 13H, 1070 16 belonging to the Hungarian clone B was detected in seven (21.9%) of the sequenced strains and included human and food isolates from 2010, 2013 and 2015 (**Figure 3**). The *bla*_CTX-M-65_ harboring isolates 21-13, 144-13 and 125-15 were more distantly related to the isolates that clustered with the Hungarian clone B. All three isolates were closely related to, and formed a distinct cluster with the *bla*_CTX-M-65_ harboring human isolate (italy 2014) from Italy and the food isolate CFSAN 042077 from the United States.

Four (12.5%) of the genomes (isolates 15-15, 169-10, 112-13 and 133-13, respectively) grouped in a distinct cluster with non-*bla*_ESBL_, pESI-like harboring strains isolated previously from broiler meat in Italy. One environmental isolate (isolate 70-15) originating from Israel formed a cluster with three strains from Israel (pESI containing 120100 and 119944, and CFSAN014765). Two fully susceptible isolates (63-13 and 126-15) were placed in the phylogenetic tree together with the non-pESI containing strain 335 3 isolated in Israel in 1971 (**Figure 3**).

### Accession Numbers

The accession numbers of the 32 sequenced isolates are listed in **Table 3** and Supplementary Tables S1–S3. This Whole Genome Shotgun project has been deposited at DDBJ/ENA/GenBank under the accession NAOX00000000. The version described in this paper is version NAOX00000000.1.

## Discussion

*S*. serovar Infantis has emerged as an important disseminator of MDR in the food chain, representing a threat to human health (Nógrády et al., 2012). The majority of strains in this study exhibited either the NaSuT pattern or the characteristic NaSSuT pattern previously identified in the MDR *S*. Infantis clone B that emerged in broilers in farms in Hungary and subsequently spread within and outside of Europe, including to the countries of Poland, Austria, Germany, Israel and Japan (Hauser et al., 2012; Nógrády et al., 2012). WGS performed in this study detected clonality in strains deemed different by PFGE and provides evidence that the Hungarian clone B has persisted since at least 2010 in Switzerland within the food chain and is associated with human disease. Strains belonging to three other closely related clusters were less prevalent but showed similar resistance patterns and persistence within food and human isolates.

In addition, we observed the occurrence of *S*. serovar Infantis harboring *bla*_CTX-M-65_. This *bla* gene has been detected previously in one human *S*. serovar Infantis isolate in Great Britain and in one from Italy (Burke et al., 2014), and furthermore, in an outbreak of *S*. Infantis in Ecuador (Cartelle Gestal et al., 2016), as well as in food in the United States. The results presented in this study show that the *bla*_CTX-M-65_ harboring *S*. serovar Infantis from Switzerland belong to a unique lineage but are similar to the strains from Italy and the United States, suggesting the emergence of a *bla*_CTX-M-65_ harboring, MDR *S.* serovar Infantis lineage in Europe as well as in North and South America. Moreover, this study shows that this lineage has been present in food and humans at least as early as 2013 in Europe.

Whole genome sequencing of these strains indicate the presence of a ∼320 kb plasmid similar to the pESI plasmid, a megaplasmid carrying multiple resistance and virulence genes originally detected in *S*. serovar Infantis in Israel in 2008 (Aviv et al., 2014). In addition, resistance genes *drfA14* and *fosA* (trimethoprim and phenicol resistance genes, respectively) which are also found on pESI-like plasmids (Franco et al., 2015), were detected in all *bla*_CTX-M-65_ harboring strains. Several other non-*bla*_ESBL_ harboring strains from this study had a close phylogenetic-relatedness to strains from Israel and Italy which harbor non-*bla*_ESBL_ pESI or pESI-like plasmids (Aviv et al., 2014), suggesting that certain *S*. serovar Infantis clones or lineages are acquiring these plasmids independently.

Our results correlate with the recent detection of a clone harboring a pESI-like plasmid and the *bla*_CTX-M-1_ gene in the broiler industry and humans in Italy (Franco et al., 2015), and suggest that some *S*. serovar Infantis clones harboring pESI-like plasmids may be undergoing a microevolution by acquiring *bla*_ESBL_ genes. While in Europe *bla*_CTX-M-1_ is prevalent within the poultry industry and has been well documented (Zurfluh et al., 2014), *bla*_CTX-M-65_ has rarely been described. By contrast, it is a prevalent *bla*_ESBL_ gene in animal and human *E. coli* strains and *S*. serovar Indiana isolates in China (Bai et al., 2016), from where it may have disseminated via horizontal transfer. However, further studies are needed to clarify the origins of this *bla*_ESBL_ gene and to characterize and compare the plasmids carrying *bla*_ESBL_ genes in *S*. serovar Infantis.

The emergence of MDR *S*. Infantis with resistance to third generation cephalosporins in food and in humans is of great concern, particularly since these strains show intermediate resistance to ciprofloxacin. The use of fluoroquinolones for the treatment of infections caused by such strains may be associated with unfavorable treatment outcomes and the selection of high-level ciprofloxacin resistance (Humphries et al., 2012).

This study extends our knowledge on clones of *S*. serovar Infantis circulating in food and causing disease in humans and provides evidence for the emergence of an MDR, ESBL-producing clone harboring *bla*_CTX-M-65_ in Switzerland. Our results highlight the necessity of strategies to reduce the prevalence of *S*. serovar Infantis within the food producing industry.

## Author Contributions

DH, BT, and RS designed the study. KZ, DH, and DA carried out the microbiological and molecular biological tests. GG, HC, FN, and BT carried out whole genome sequencing. MN-I, RS, DH, GG, and BT analyzed and interpreted the data. MN-I drafted the manuscript. All authors read and approved the final manuscript.

## Conflict of Interest Statement

The authors declare that the research was conducted in the absence of any commercial or financial relationships that could be construed as a potential conflict of interest.

## Footnotes

1 http://www.ncbi.nlm.nih.gov/blast/

2 http://www.cdc.gov/pulsenet/protocols.htm

3 https://cge.cbs.dtu.dk/services/MLST/

4 https://cge.cbs.dtu.dk/services/ResFinder/

5 https://cge.cbs.dtu.dk/services/PlasmidFinder/
